# Automated radiolabelling of [^68^Ga]Ga-PSMA-11 (gallium (^68^Ga)-gozetotide) using the Locametz® kit and two generators

**DOI:** 10.1186/s41181-024-00260-4

**Published:** 2024-04-17

**Authors:** Elke A. van Brandwijk, Else A. Aalbersberg, Arman S. Hosseini, Alwin D. R. Huitema, Jeroen J. M. A. Hendrikx

**Affiliations:** 1 Department of Nuclear Medicine, The Netherlands Cancer Institute, Plesmanlaan 121, 1066 CX Amsterdam, The Netherlands; 2 Department of Pharmacy and Pharmacology, The Netherlands Cancer Institute, Amsterdam, The Netherlands; 3 Department of Clinical Pharmacy, University Medical Centre Utrecht, Utrecht University, Utrecht, The Netherlands; 4 Department of Pharmacology, Princess Máxima Centre for Pediatric Oncology, Utrecht, The Netherlands

**Keywords:** Gallium-68, PSMA, [^68^Ga]Ga-PSMA-11, PSMA-11, Prostate cancer, Automated synthesis, Quality control, Locametz®

## Abstract

**Background:**

Steps have been taken by pharmaceutical companies to obtain marketing authorisation of PSMA ligands in the European Union. Since December 2022, Locametz® (PSMA-11, gozetotide) is licensed as kit for manual radiolabelling with gallium-68 and commercially available since mid-2023. The Summary of Product Characteristic (SmPC) describes manual radiolabelling with a maximum activity after radiolabelling of 1369 MBq. We aimed for radiolabelling with a higher activity to increase production efficiency, and thus, automated radiolabelling is strongly preferred over manual radiolabelling to reduce radiation exposure to personnel. The aim of this study was to develop and validate a method for automated radiolabelling of the Locametz® kit using ~ 2000 MBq of gallium-68 eluate for radiolabelling.

**Results:**

Automated radiolabelling of [^68^Ga]Ga-PSMA-11 using the Locametz® kit provided a product which complies to the Ph. Eur., had a shelf-life of 6 h at room temperature, and theoretically reduced radiation exposure 5.7 times. Radiolabelling with one and two generator(s) resulted in a radiochemical yield of 91–102% and 96–101% after preparation, respectively. The radiochemical purity ranged from 98.0 to 99.6% for radiolabelling with one generator and ranged from 98.4 to 99.3% for radiolabelling with two generators with similar stability. The activity of the final product was much higher when using two generators, 1961–2035 MBq compared to 740–1260 MBq, which leads to ~ 1.5 times more patient syringes available per preparation.

**Conclusion:**

Automated radiolabelling of [^68^Ga]Ga-PSMA-11 using the Locametz® kit with higher gallium-68 activity than specified in the SmPC results in a product that is in compliance with the Ph. Eur. monograph and has a shelf-life of 6 h at room temperature. Radiolabelling with two generators proved possible and resulted in a product with similar quality but with much higher efficiency.

**Supplementary Information:**

The online version contains supplementary material available at 10.1186/s41181-024-00260-4.

## Background

In 2022 more than 470,000 men were diagnosed with prostate cancer in Europe (World Health Organization 2024). For diagnosis and therapy, prostate specific membrane antigen (PSMA) is used as a target as it is a type II membrane protein which is expressed in prostate tissue and in prostate carcinomas (Fendler et al. 2023). Gallium-68 labelled Glu-urea-Lys(Ahx)-HBED-CC ([^68^Ga]Ga-PSMA-11, Gallium (^68^Ga)-gozetotide) is a radiolabelled PSMA inhibitor used to visualize prostate tumours through positron emission tomography/computed tomography (PET/CT) (Eder et al. 2014).

In the past years, PSMA PET/CT diagnostic imaging has developed as a proven technique for the staging of metastasized prostate carcinoma (Fendler et al. 2023). Initially, [^68^Ga]Ga-PSMA-11 was prepared as an in-house produced radiopharmaceutical which was an alternative for choline products (Afshar-Oromieh et al. 2014). Later, more variants including ^18^F-ligands such as [^18^F]F-DCFPyl ([^18^F]-piflufolastat; Pylclari®) and [^18^F]F-PSMA-1007 (Radelumin®) have been developed (Szabo et al. 2015; Giesel et al. 2017; ABX 2024). Steps have been taken by pharmaceutical companies to obtain marketing authorisation of PSMA ligands in the European Union. PSMA-11 (gozetotide, Locametz®) is licensed as kit for radiolabelling with gallium-68. Key point in the regulation of medicinal products, including radiopharmaceuticals, in the European Union is securing patient safety and product quality. This is regulated by marketing authorisation of medicinal products and licenses for manufacturing and wholesale of these (Scheepers et al. 2017). Based on the same directive, in-house (radio)pharmacy preparations (as Magistral Formula or Officinal Formula) are allowed to fulfil unmet needs of patients and facilitate fast implementation of new diagnostic radiopharmaceuticals (Scheepers et al. 2017; Hendrikse et al. 2022). The in-house preparation of [^68^Ga]Ga-PSMA-11 enabled fast introduction of PSMA PET/CT. However, as marketing authorisation is the preferred pathway, the authorisation of the Locametz® kit requires reconsideration of the in-house preparation (Scheepers et al. 2017; Hendrikse et al. 2022; Korde et al. 2024).

In December 2022 the Locametz® kit was licensed in the European Union for the preparation of [^68^Ga]Ga-PSMA-11 and since June 2023 the kit is commercially available in the Netherlands (EMA 2023). The Summary of Product Characteristic (SmPC) of the Locametz® kit describes manual radiolabelling with a maximum activity after radiolabelling of 1369 MBq (EMA 2023). To increase production efficiency higher activity than described in the SmPC should be investigated. However, for hospitals using automated radiolabelling for in-house production, manual radiolabelling will result in seven times higher radiation exposure for the operator making automated radiolabelling safer for personnel (Kleynhans et al. 2020). When using higher activity than described in the SmPC and the development of new germanium-68/gallium-68 (^68^Ge/^68^Ga) generators with higher activity in each elution make reduction of radiation exposure to the operator even more important (Waterhouse et al. 2020; FDA has issued Drug Master File for ITM’s Gallium Generator GeGant®. 2023; IRE ELiT radiopharma 2023). Besides lower radiation exposure, automated radiolabelling provides robust and repeatable processes which makes the radiolabelling more reliable and less prone to inter-individual variation between operators (Kleynhans et al. 2020; Meisenheimer et al. 2020; Nelson et al. 2022). Because automated radiolabelling is used, the preparation can be performed with higher activity to obtain more patient syringes from one kit.

The aim of this study was to develop and validate a method for automated radiolabelling of the Locametz® kit using ~ 2000 MBq of gallium-68 eluate for radiolabelling.

## Methods

Firstly, the synthesis program for automated radiolabelling was developed and the sequence of operations was tested in non-radioactive runs. Secondly, the developed program was used to prepare [^68^Ga]Ga-PSMA-11 with the Locametz® kit in a test run, after which the synthesis program was locked. Finally, the production process using the locked synthesis program was validated by testing the final product according to specifications based on the European Pharmacopoeia (Ph. Eur.) monograph 04/2021:3044: identification, appearance, pH, sterility, endotoxin, non-complexed gallium-68 species, radiochemical purity, and germanium-68 breakthrough (European Pharmacopoeia 2023).

### Materials

Locametz® kit (EU/1/22/1692/001 gozetotide, 25 µg, kit for radiopharmaceutical preparation) was obtained from Novartis Pharma B.V. (Amsterdam, Netherlands). IRE ELiT GalliAd® 1.85 GBq ^68^Ge/^68^Ga generators were obtained from IRE (Fleurus, Belgium). The cassette (IND-0015) and Ga-PSMA-11 (non-radioactive reference standard) were purchased from ABX (Radeberg, Germany). Sodium chloride (NaCl) 0.9% solution was obtained from B Braun (Melsungen, Germany). Acetonitrile > 99.9% and water for injections (UPLC/MS) were obtained from Biosolve BV (Valkenswaard, The Netherlands), trifluoroacetic acid ≥ 99.8% (TFA) from Merck Millipore (Darmstadt, Germany), and methanol (Ph. Eur. Quality) and ammonium acetate ≥ 98% were obtained from Sigma Aldrich, Merck (Darmstadt, Germany).

### Automated radiolabelling with Scintomics® GRP module

**Fig. 1:**
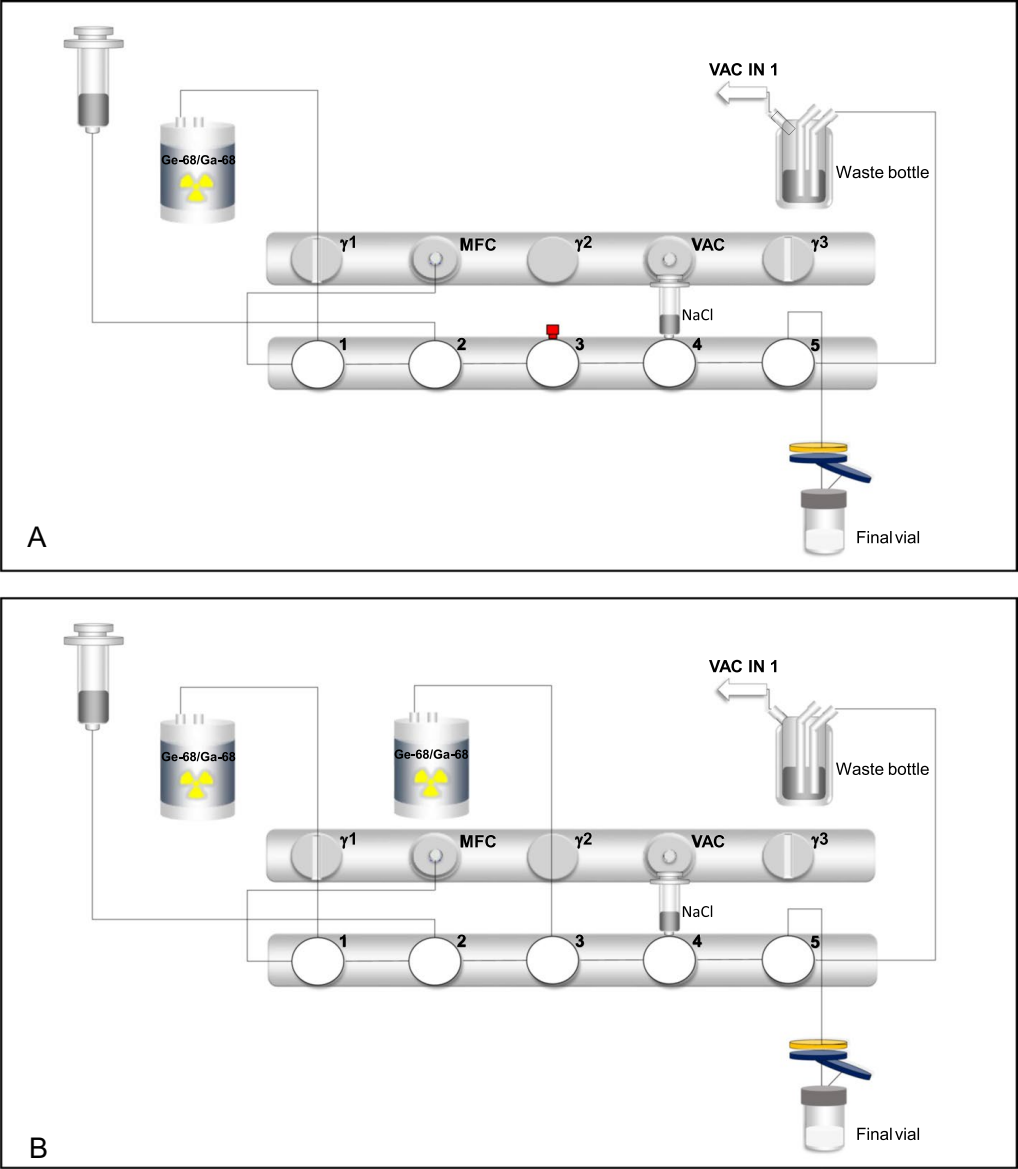
Visualization scheme of the automated Scintomics® system for radiolabelling of [^68^Ga]Ga-PSMA-11. **A** Using one generator and **B** using two generators. The scheme represents the connections made to the manifold and the placing of sodium chloride syringe. The red cap at position 3 in **A** represents an universal luer lock stopper (male/female connection). The syringe in position 4 contains 8 mL NaCl in **A** and 7 mL NaCl in **B**. MFC, mass flow controller; VAC, vacuum; NaCl, sodium chloride; Ge-68/Ga-68, germanium-68/gallium-68

Automated radiolabelling and dilution was performed using the eluate of one or two generator(s) with a Scintomics® GRP 3V module (Scintomics® GmbH, Fürstenfeldbruck, Germany). The set-up of the manifold was based on the instructions for preparation described in the SmPC of Locametz® (EMA 2023). Two different programs were developed: one for the radiolabelling in compliance with the SmPC (eluate of one generator) and one using higher activity (eluate of two generators) (see Additional files 1 and 2). A schematic overview of the system used is presented in Fig. 1A and B for the one and two generator(s) configuration, respectively.

Based on the steps in the SmPC of Locametz®, a time control file (TCF) was developed and a step for elution of the second generator was added. This leads to the following steps of our method: (1) eluate of the first generator is added to the Locametz® vial (with sterile filtration via a 0.22 µm filter). (2) Eluate of the second generator is added to the Locametz® vial through the same process as the first generator. (3) Five minutes incubation at room temperature. (4) Dilution with NaCl 0.9% to a final volume of 9.2 mL.

### Quality controls

#### Activity and radiochemical yield

The activity of the produced [^68^Ga]Ga-PSMA-11 was measured in a dose calibrator type VIK-202-5051 (Comecer Netherlands, Joure, the Netherlands). To determine the radiochemical yield, the theoretical activity was first calculated. The theoretical activity of [^68^Ga]GaCl_3_ was calculated using the calculation described in the SmPC of the GalliAd® and is shown in Eq. 1 (IRE ELiT 0.^74^–^1^.^85^ GBq, radionuclide generator 2023). The radiochemical yield was estimated using the theoretical activity of [^68^Ga]GaCl_3_ obtained from the generator and the activity of the produced [^68^Ga]Ga-PSMA-11. The activities of germanium-68 and gallium-68 were corrected for decay.

1 $$Theoretical\;activity\;out\;of\;the\;generator\;(MBq) = activity\;of\;the\;generator\;(MBq) \times decay\;factor \times build\;up\;factor \times 0.70$$

#### Non-complexed gallium-68 species

To determine the non-complexed gallium-68 species (e.a. gallium-68 ions and gallium-68 colloids) instant thin layer chromatography (iTLC) was used. The stationary phase consisted of iTLC-silica gel (iTLC-SG) glass fibre strip (Agilent Technologies, Santa Clara, USA) and the mobile phase of methanol/1 M ammonium acetate (50:50 v/v). After applying 5 µL of [^68^Ga]Ga-PSMA-11 on the iTLC-SG strip, the strip was placed in the mobile phase. When the solvent front had moved 6 cm the strip was read with the Radio-TLC scanner ScanRAM Model 1E with LAURA software (LabLogic Systems Ltd., Sheffield, UK). Non-complexed gallium-68 species should be ≤ 3% of the total activity in accordance with Ph. Eur. monograph 04/2021:3044 (European Pharmacopoeia 2023).

#### Identification and radiochemical purity

Identification and radiochemical purity of [^68^Ga]Ga-PSMA-11 was performed with high performance liquid chromatography (HPLC). HPLC was performed with a Dionex ultimate 3000 UHPLC system coupled to a Dionex 3000 ultraviolet (UV) detector (ThermoFisher Scientific, Waltham, USA) and a Canberra Packard flow scintillation analyser with a gamma cell (Canberra Packard GmbH, Schwadorf, Austria). Eluent A consisted of 0.1% TFA in water and eluent B consisted of 0.1% TFA in acetonitrile. At t = 0 and t = 2 h samples were diluted 100-fold in water, at t = 4 h the sample was diluted tenfold in water, and at t = 6 h the sample was not diluted such that the activity in all samples were within the validated linear range of the gamma detector. The samples were analysed with a reversed phase C18 column (4.6 × 250 mm, 5 µm) (Waters Symmetry Shield) and linear gradient of 90% A and 10% B to 10% A and 90% B in 8 min followed by 2 min of 90% A and 10% B with a flow rate of 1.5 mL/min. The retention time of the reference standard (non-radioactive Ga-PSMA-11) should be between 4.5 and 5.5 min. The difference in retention time of [^68^Ga]Ga-PSMA-11 and the reference standard should be 0.4 ± 0.2 min. The retention time of Ga-PSMA-11 was measured with HPLC with UV detection and the retention time of [^68^Ga]Ga-PSMA-11 with the gamma detector which have been serially coupled which causes the difference in time.

Radiation detection was performed between 300 and 600 keV. The peak area of [^68^Ga]Ga-PSMA-11 should be > 95% of the total area of all peaks in accordance with the Ph. Eur. monograph 04/2021:3044 (European Pharmacopoeia 2023). Since HPLC does not detect colloids, the radiochemical purity based on HPLC has to be corrected. Gallium-68 colloids were calculated by subtracting gallium-68 ions (determined with HPLC) from non-complexed gallium-68 species (determined with iTLC). Radiochemical purity was calculated by subtracting gallium-68 colloids from [^68^Ga]Ga-PSMA-11.

#### Appearance and pH

The appearance of the final product should be clear, colourless, and without undissolved matter which was determined with visual inspection. The pH was measured with a pH indicator strip (Merck Millipore, Darmstadt, Germany) and determined with a pH test strip reader Quantofix Relax (Macherey–Nagel GmbH & Co. Kg, Düren, Germany) and should lie between 3.2 and 6.5 in accordance with the SmPC of Locametz® (EMA 2023).

#### Sterility and endotoxins

To determine sterility, 5.0 mL of [^68^Ga]Ga-PSMA-11 was added to 10 mL tryptic soy broth (TSB) medium (Biotrading Benelux B.V., Mijdrecht, The Netherlands) and incubated for 14 days at 30–35 °C. The TSB medium should be clear after the incubation period. Endotoxins were determined according to Ph. Eur. monograph 01/2018:20614 by using the Endosafe nexgen-PTS™ (Charles River Laboratories, Ecully, France). The limit of endotoxins is < 32 IU/mL (based on a dose of 300 MBq in 5.5 mL). The limit was calculated using the limit of < 175/V IU/mL (V being the maximum recommended dose in millilitres) in accordance with Ph. Eur. monograph 04/2021:3044 (European Pharmacopoeia 2023).

#### Germanium-68 breakthrough

After 48 h germanium-68 breakthrough was measured with gamma-ray spectrometry (Canberra, Zellik, Belgium). The sample was diluted 10,000-fold and was analysed at 511 keV using the multichannel analyser (Canberra, Zellik, Belgium). The measured activity should be < 0.001% of the total activity measured at t = 0 h in accordance with Ph. Eur. monograph 04/2021:3044 (European Pharmacopoeia 2023).

#### Validation and stability

The validation was performed with three preparations for each method on three separate days. All quality controls were performed at t = 0 h. In addition appearance, pH, determination of non-complexed gallium-68 species, and determination of [^68^Ga]Ga-PSMA-11 were performed at room temperature at t = 2, t = 4, and t = 6 h to investigate the stability since more acid is added and higher activity is used than described in the SmPC of Locametz® (EMA 2023).

### Radiation safety

The radiation exposure for the automated method was compared to the radiation exposure for the method described in the SmPC. Radiation exposure was calculated using the equation for intensity of transmitted radiation (Iowa State University 2024). Time to elute gallium-68 from the generator, activity, and half-value thickness were considered equal in both methods whilst the lead equivalent in shielding during automated radiolabelling was 30 mm and 15 mm during manual radiolabelling.

Additional to the calculation of the theoretical radiation exposure, the actual radiation exposure and dose rate during automated radiolabelling were measured for the worst case scenario (use of two generators) at a distance of 0 cm and 30 cm from the lead shielding during three preparation runs. The radiation exposure was measured using DMC 3000™ Personal Electronic Dosimeter and the dose rate was measured using FH 40 G Multi-Purpose Digital Survey Meter.

## Results

### Method development

To automatically elute gallium-68 from the generator, the generator is coupled to the manifold and eluted by creating a vacuum with the 20 mL syringe in the syringe pump of the Scintomics® module after starting the synthesis program. This reduces the risk of vacuum failure since the vacuum is no longer dependent on needle placement. The automated dilution step is performed directly after synthesis using the module instead of doing it manually, which reduces radiation exposure and the risk of contamination. The TCF is provided as attachment.

### Automated radiolabelling

Three automated radiolabelling runs were completed successfully for both the radiolabelling with gallium-68 activity corresponding to the SmPC and higher gallium-68 activity. All results met the acceptance criteria for the final product. Based on radiation safety calculations, automated radiolabelling provides 5.7 times lower radiation exposure than the method described in the SmPC due to more lead equivalent in shielding during automated radiolabelling. Time was not considered as a factor in the calculations since preparation time was considered to be similar for both methods. During automatic radiolabelling using the eluate of two generators, the measured dose rate ranged from 0.12 to 22.6 μSv/h at a distance of 0 cm from the lead shielding and 0.04 to 9.72 μSv/h at a distance of 30 cm from the lead shielding. The average radiation exposure was 3.27 μSv (3.0–3.56 μSv) at a distance of 0 cm from the lead shielding and 0.46 μSv (0.37–0.51 μSv) at a distance of 30 cm from the lead shielding.

The results for validation runs using one and two generator(s) are shown in Tables 1 and 2, respectively. The radiochemical yield of both radiolabelling runs were comparable (91–102% and 96–101%). The pH of the product with higher activity was slightly lower (4.6–4.7 vs 4.9–5.1). The radiolabelling process with the eluate of one or two generator(s) both met the acceptance criteria for non-complexed gallium-68 species. A shelf-life of 6 h at room temperature was demonstrated, regardless of using the eluate of one or two generators.

**Table 1:** Results of three validation batches of [^68^Ga]Ga-PSMA-11 using eluate of one generator

Item	Time (h)	Batch 1	Batch 2	Batch 3
Age generator (weeks)		3	25	26
Radiochemical yield (%) (Corrected for decay)	*T* = *0*	102	92	91
Gallium-68 activity (MBq)	*T* = *0*	1260	763	740
Identification (min) (Criteria: Rt_68Ga-PSMA-11_–Rt_ref_ = 0.4 ± 0.2 min)	*T* = *0*	0.39	0.39	0.36
Appearance (Criteria: clear, colourless, and without undissolved matter)	*T* = *0*	Complies	Complies	Complies
	*T* = *2*	Complies	Complies	Complies
	*T* = *4*	Complies	Complies	Complies
	*T* = *6*	Complies	Complies	Complies
pH (Criteria: pH 3.2–6.5)	*T* = *0*	4.9	4.9	5.0
	*T* = *2*	4.9	5.0	4.9
	*T* = *4*	5.0	5.1	4.9
	*T* = *6*	5.0	5.1	5.0
Sterility (Criteria: TSB clear after 14-day incubation)		Complies	Complies	Complies
Endotoxin (IU/mL) (Criteria: < 32 IU/mL)		< 0.050	< 0.050	< 0.050
Non-complexed gallium-68 species (%) (Criteria: ≤ 3%)	*T* = *0*	0.47	0.49	0.60
	*T* = *2*	0.47	0.64	0.58
	*T* = *4*	0.78	0.52	0.57
	*T* = *6*	0.82	0.95	0.97
Radiochemical purity (%) (Criteria: [^68^Ga]Ga-PSMA-11 > 95%)	*T* = *0*	99.2	99.3	99.2
	*T* = *2*	99.6	98.0	98.8
	*T* = *4*	99.1	99.3	99.3
	*T* = *6*	99.1	99.0	99.0
Germanium-68 breakthrough (%) (Criteria: < 0.001%)	*T* = *0*	0.00008	0.00002	0.00006

Rt, retention time; ref, reference; TSB, tryptic soy broth

**Table 2:** Results of three validation batches of [^68^Ga]Ga-PSMA-11 using eluate of two generators

Item	Time (h)	Batch 4	Batch 5	Batch 6
Age generator (weeks)		Generator 1: 2 Generator 2: 27	Generator 1: 2 Generator 2: 28	Generator 1: 3 Generator 2: 28
Radiochemical yield (%) (Corrected for decay)	*T* = *0*	96	99	101
Gallium-68 activity (MBq)	*T* = *0*	1961	2018	2035
Identification (min) (Criteria: Rt_68Ga-PSMA-11_–Rt_ref_ = 0.4 ± 0.2 min	*T* = *0*	0.38	0.38*	0.38
Appearance (Criteria: clear, colourless, and without undissolved matter)	*T* = *0*	Complies	Complies	Complies
	*T* = *2*	Complies	Complies	Complies
	*T* = *4*	Complies	Complies	Complies
	*T* = *6*	Complies	Complies	Complies
pH (Criteria: pH 3.2–6.5)	*T* = *0*	4.7	4.6	4.6
	*T* = *2*	4.6	4.6	4.6
	*T* = *4*	4.6	4.6	4.6
	*T* = *6*	4.6	4.6	4.6
Sterility (Criteria: TSB clear after 14-day incubation)		Complies	Complies	Complies
Endotoxin (IU/mL) (Criteria: < 32 IU/mL)		< 0.050	< 0.050	< 0.050
Non-complexed gallium-68 species (%) (Criteria: ≤ 3%)	*T* = *0*	0.73	0.96	1.12
	*T* = *2*	0.69	0.77	0.74
	*T* = *4*	0.62	0.70	0.67
	*T* = *6*	0.67	0.88	1.11
Radiochemical purity (%) (Criteria: [^68^Ga]Ga-PSMA-11 > 95%)	*T* = *0*	99.1	–**	98.4
	*T* = *2*	99.0	99.0	99.0
	*T* = *4*	99.2	99.1	99.2
	*T* = *6*	99.3	99.0	98.8
Germanium-68 breakthrough (%) (Criteria: < 0.001%)	*T* = *0*	0.00009	0.00009	0.00007

Rt, retention time; ref, reference; TSB, tryptic soy broth

*Determined at t = 2 h due to technical issues at t = 0 h

**No data available due to technical issues

Despite the adjustments to the instructions in the SmPC, the final product still complies to the Ph. Eur. monograph 04/2021:3044 and is therefore suitable for clinical use. Moreover, automatic radiolabelling provides 5.7 times less radiation exposure than the manual method.

### Radiolabelling using higher activity

The gallium-68 activity in the final product vial at the end of synthesis ranged from 740 to 1260 MBq using gallium-68 activity corresponding with the SmPC and from 1961 to 2035 MBq using higher gallium-68 activity. Note that generators of a different age were used, see Tables 1 and 2. The gallium-68 activity of the final product vial is much higher when using two generators, which leads to ~ 1.5 times more patient syringes in comparison to the method described in the SmPC, see Table 3.

**Table 3:** Comparison of the number of patient syringes derived after preparation using both radiolabelling methods

Type of scanner	Number of patient syringes derived SmPC preparation*	Number of patient syringes derived Current method*
1 conventional PET/CT	2	3
2 conventional PET/CT in parallel	3	5
1 long axial field of view (LAFOV) PET/CT	7	10

*Calculations were based on a required activity of [^68^Ga]Ga-PSMA-11 of 2 MBq/kg (average weight is 80 kg) when using conventional PET/CT and 50 MBq when using LAFOV PET/CT (EMA 2023). Time of positioning and scanning of a patient was assumed to be 45 min for conventional PET/CT and 15 min for LAFOV PET/CT

## Discussion

The present study demonstrates that automated radiolabelling of [^68^Ga]Ga-PSMA-11 using the Locametz® kit results in a product that is in compliance with the Ph. Eur. monograph and has a shelf-life of 6 h at room temperature. Radiolabelling using more gallium-68 activity than described in the SmPC is as stable as the product described in the SmPC.

### Automated radiolabelling using higher activity

The preparation described in the SmPC of Locametz® uses one generator to radiolabel [^68^Ga]Ga-PSMA-11 (EMA 2023). The present study demonstrates that the kit can be used with the eluate of two generators, and thus providing higher gallium-68 activity: the gallium-68 activity in the final product using the eluate of two generators was 1.5 to 2.5 times higher than after using the eluate of one generator (actual difference was depending on the age of the generators). The quality of the product using both methods was similar, with only the pH being different. The pH of the preparation after radiolabelling with higher gallium-68 activity was slightly lower (4.6–4.7 vs 4.9–5.1) due to addition of double the amount of hydrochloric acid (HCl) compared to radiolabelling in compliance with the SmPC of Locametz® (1.1 mL vs 2.2 mL 0.1 M HCl). Although the pH is slightly lower, the radiolabelling process was not affected and the pH of the final product was within the range of the acceptance criteria for intravenously administered bolus solutions. This was expected since the Locametz® kit can also be used with the GalliaPharm® ^68^Ge/^68^Ga-generator which is eluted with 5 mL 0.1 M HCl, indicating that the kit excipients are equilibrated to at least 5 mL of HCl (EMA 2023; HRPA 0.^74^–^1^.^85^ GBq radionuclide generator 2023). However, this also indicates that the presented approach of radiolabelling with two generators might not be feasible with two GalliaPharm® generators.

### Locametz® kit versus other PSMA-11 kits

Currently, the Locametz® kit is the only kit with marketing authorisation available in Europe and thus the preferred kit from a regulatory point of view. However, more kits with a European marketing authorisation might be available in the near future. Illuccix® is a PSMA-11 kit which currently has marketing authorisation in the United States, Australia, and New Zealand (Telix 2024). The kit is prepared without purification and at room temperature, which is similar to the Locametz® kit, but the PSMA-11 first has to be dissolved in an acetate buffer before adding it to [^68^Ga]GaCl_3_ (Telix Pharma 2024). The IsoPROtrace® kit is commercially available and expected to obtain marketing authorisation in the half of 2024. Like the Locametz® kit, the IsoPROtrace® kit consists of a single vial for radiolabelling, however, the amount of PSMA-11 in the vial (i.e. vial content) is less (10 μg vs 25 μg) (Isotopia Nuclear Medicine 2024). Further test are required to see if radiolabelling with the eluate of two generators is feasible with the IsoPROtrace® kit. Once other kits with marketing authorisation become available, one should balance advantages (e.g. radiolabelling with higher activity, single vial radiolabelling, costs) against disadvantages (e.g. higher risk for microbiological contamination with multiple process steps) to select the best local option. On forehand, no specific kit is preferred over other.

### Method transfer to other radiopharmacies

Implementation of automated radiolabelling of [^68^Ga]Ga-PSMA-11 with the Locametz® kit in other radiopharmacies using the Scintomics® module is most likely fast when using the provided TCF and only requires local implementation of the automated process. This can be reduced to a single validation run for correct implementation based on a risk assessment (Aerts et al. 2014). When using another module than the Scintomics® module, a new control program should be developed based on the steps described in the method development section, which requires a full validation of the new sequence to assure local compliance of the radiolabelling to the Ph. Eur. monograph (Gillings et al. 2021).

## Conclusions

Automated radiolabelling of [^68^Ga]Ga-PSMA-11 using the Locametz® kit and higher gallium-68 activity than specified in the SmPC of Locametz® results in a product that is in compliance with the Ph. Eur. monograph and has a shelf-life of 6 h at room temperature, which is approximately six times the half-life for physical decay of gallium-68. Overall, radiolabelling with two generators proved possible and resulted in a product with similar quality but with a much higher efficiency.

### Supplementary Information

**Additional file 1.** Time control file for radiolabelling in compliance with the SmPC.

**Additional file 2.** Time control file for radiolabelling using higher activity (eluate of two generators).

## Data Availability

The datasets used and/or analysed during the current study are available from the corresponding author on reasonable request.

## Abbreviations

[^68^Ga]Ga-PSMA-11: Gallium-68 labelled Glu-urea-Lys(Ahx)-HBED-CC
^68^Ge/^68^Ga: Germanium-68/gallium-68
HCl: Hydrochloric acid
HPLC: High performance liquid chromatography
iTLC: Instant thin layer chromatography
iTLC-SG: Instant thin layer chromatography silica gel
NaCl: Sodium chloride
PET/CT: Positron emission tomography/computed tomography
Ph. Eur.: European Pharmacopoeia
PSMA: Prostate specific membrane antigen
Ref: Reference
Rt: Retention time
SmPC: Summary of Product Characteristic
TCF: Time control file
TSB: Tryptic soy broth
UV: Ultraviolet
