# Supplementary material for: Automated radiolabelling of [68Ga]Ga-PSMA-11 (gallium (68Ga)-gozetotide) using the Locametz® kit and two generators
Source: EJNMMI Radiopharm Chem. 2024 Apr 17;9:31. doi: 10.1186/s41181-024-00260-4 (PMC11024066; doi:10.1186/s41181-024-00260-4)
Supplement: Supplementary file 1 — Additional file 1. Time control file for radiolabelling in compliance with the SmPC. [file 41181_2024_260_MOESM1_ESM.docx]

| **Time [min]** | **Function** | **Parameter** |
| --- | --- | --- |
| 0.03 | Display information | “Elution generator” |
| 0.04 | Start Chromatogram | Ch. 4,7,8,9 (500 ms) |
| 0.05 | Valve position | Valve 2 = position 4 |
| 0.06 | Valve position | Valve 1 = position 2 |
| 0.13 | Dispenser (Dispenser 1) | Aspirates 17000 µl from 1 (50.000 µl/min) |
| 3.10 | Wait for Input Signal | Dispenser 1 ready |
| 3.11 | Valve position | Valve 1 = Position 3 |
| 3.18 | Dispenser (Dispenser 1) | Aspirates 2000 µl from 1 (75000 µl/min) |
| 3.26 | Wait for Input Signal | Dispenser 1 ready |
| 3.27 | Display information | “Transfer activity into vial” |
| 3.28 | Valve position | Valve 5 = Position 4 |
| 3.29 | Valve Position | Valve 2 = Position 2 |
| 3.36 | Dispenser (Dispenser 1) | Dispenser 1 Dispenses 19000 µl to 1 (142500 µl/min) |
| 4.06 | Wait for Input Signal | Dispenser 1 ready |
| 4.07 | Display Information | “Add NaCl” |
| 4.08 | Valve Position | Valve 4 = Position 4 |
| 4.15 | Dispenser (Dispenser 1) | Aspirates 15000 µl from 1 (50000 µl/min) |
| 9.15 | Wait for Input Signal | Dispenser 1 ready |
| 9.16 | Valve Position | Valve 4 = Position 3 |
| 9.17 | Display information | “Transfer NaCl into vial” |
| 9.23 | Dispenser (Dispenser 1) | Dispenser 1 Dispenses 15000 µl to 1 (142500 µl/min) |
| 9.58 | Wait for Input Signal | Dispenser 1 ready |
| 9.59 | Valve position | Valve 2 = Position 3 |
| 10.00 | Valve position | Valve 5 = Position 3 |
| 10.07 | Valve position | Vacuum Exhaust = open |
| 10.17 | Valve position | Vacuum Exhaust = closed |
| 10.18 | Stop Chromatogram | Channel 4,7,8,9 |
| 10.19 | Stop all |  |
