# Supplementary material for: Automated radiolabelling of [68Ga]Ga-PSMA-11 (gallium (68Ga)-gozetotide) using the Locametz® kit and two generators
Source: EJNMMI Radiopharm Chem. 2024 Apr 17;9:31. doi: 10.1186/s41181-024-00260-4 (PMC11024066; doi:10.1186/s41181-024-00260-4)
Supplement: Supplementary file 2 — Additional file 2. Time control file for radiolabelling using higher activity (eluate of two generators). [file 41181_2024_260_MOESM2_ESM.docx]

| **Time [min]** | **Function** | **Parameter** |
| --- | --- | --- |
| 0.03 | Display information | “Elution generator 1” |
| 0.04 | Start Chromatogram | Ch. 4,7,8,9 (500 ms) |
| 0.05 | Valve position | Valve 2 = position 4 |
| 0.06 | Valve position | Valve 1 = position 2 |
| 0.13 | Dispenser (Dispenser 1) | Aspirates 17000 µl from 1 (50.000 µl/min) |
| 3.10 | Wait for Input Signal | Dispenser 1 ready |
| 3.11 | Valve position | Valve 1 = Position 3 |
| 3.18 | Dispenser (Dispenser 1) | Aspirates 2000 µl from 1 (75000 µl/min) |
| 3.26 | Wait for Input Signal | Dispenser 1 ready |
| 3.27 | Display information | “Transfer into vial” |
| 3.28 | Valve position | Valve 5 = Position 4 |
| 3.29 | Valve Position | Valve 2 = Position 2 |
| 3.36 | Dispenser (Dispenser 1) | Dispenses 19000 µl to 1 (142500 µl/min) |
| 4.06 | Valve Position | Valve 3 = Position 4 |
| 4.09 | Wait for Input Signal | Dispenser 1 ready |
| 4.13 | Dispenser (Dispenser 1) | Aspirates 17000 µl from 1 (50000 µl/min) |
| 7.13 | Wait for Input Signal | Dispenser 1 ready |
| 7.14 | Valve Position | Valve 3 = Position 3 |
| 7.15 | Valve Position | Valve 2 = Position 4 |
| 7.22 | Dispenser (Dispenser 1) | Aspirates 2000 µl from 1 (75000 µl/min) |
| 7.30 | Wait for Input Signal | Dispenser 1 ready |
| 7.31 | Valve Position | Valve 2 = Position 2 |
| 7.32 | Display information | “Transfer into vial” |
| 7.38 | Dispenser (Dispenser 1) | Dispenses 19000 µl to 1 (142500 µl/min) |
| 7.39 | Wait for Input Signal | Dispenser 1 ready |
| 8.09 | Display Information | “Add NaCl” |
| 8.10 | Valve Position | Valve 4 = Position 4 |
| 8.17 | Dispenser (Dispenser 1) | Aspirates 15000 µl from 1 (50000 µl/min) |
| 13.11 | Wait for Input Signal | Dispenser 1 ready |
| 13.12 | Valve Position | Valve 4 = Position 3 |
| 13.16 | Display information | “Transfer NaCl into vial” |
| 13.19 | Dispenser (Dispenser 1) | Dispenses 15000 µl to 1 (142500 µl/min) |
| 13.54 | Wait for Input Signal | Dispenser 1 ready |
| 13.55 | Valve position | Valve 2 = Position 3 |
| 13.56 | Valve position | Valve 5 = Position 3 |
| 14.03 | Valve position | Vacuum Exhaust = OPEN |
| 14.13 | Valve position | Vacuum Exhaust = CLOSED |
| 14.14 | Stop Chromatogram | Channel 4,7,8,9 |
| 14.15 | Stop all |  |
